# Diagnostic value of cytokine detection in children with *Mycoplasma pneumoniae* pneumonia complicated by bacterial or viral co-infections

**DOI:** 10.1186/s12887-026-06992-3

**Published:** 2026-05-14

**Authors:** ChenYe Lin, ZhiRong Zhu, Yadong Li, JianXing Wei, ZhiNan Zhang, Yanbin He, QiuYu Tang, Di Lian

**Affiliations:** 1 Department of Respiratory, Fujian Children’s Hospital (Fujian Branch of Shanghai Children’s Medical Center), College of Clinical Medicine for Obstetrics & Gynecology and Pediatrics, Fujian Medical University, Fuzhou, 350000 China; 2 Information Center, Fujian Children’s Hospital (Fujian Branch of Shanghai Children’s Medical Center), College of Clinical Medicine for Obstetrics & Gynecology and Pediatrics, Fujian Medical University, Fuzhou, 350000 China; 3 Department of Clinical Laboratory, Fujian Children’s Hospital (Fujian Branch of Shanghai Children’s Medical Center), College of Clinical Medicine for Obstetrics & Gynecology and Pediatrics, Fujian Medical University, Fuzhou, 350000 China; 4 Zhejiang Key Laboratory of Digital Technology in Medical Diagnostics, Hangzhou, China

**Keywords:** *Mycoplasma pneumoniae* pneumonia, Co-infection, Cytokines, Interleukin, Diagnosis, Machine learning

## Abstract

**Background:**

To evaluate the diagnostic value of cytokine levels in paediatric patients with *Mycoplasma pneumoniae* pneumonia (MPP) complicated by bacterial or viral infections, to enhance early detection and treatment strategies.

**Methods:**

A retrospective analysis was conducted on 145 paediatric patients with MPP at Fujian Children’s Hospital between October 2023 and April 2024. The patients were categorised into three groups: MPP only, MPP with bacterial co-infection, and MPP with viral co-infection. Clinical and laboratory data were analysed. Diagnostic accuracy was assessed using receiver operating characteristic (ROC) curves. Machine learning models with Boruta feature selection and 5-fold cross-validation were applied for co-infection classification. Statistical significance was set at *P* < 0.05.

**Results:**

Significant differences were observed between groups. The bacterial co-infection group had higher white blood cell and platelet counts and elevated levels of interferon-alpha, interleukin-1 beta, interleukin-6, and tumour necrosis factor-alpha (all *P* < 0.05). The viral co-infection group had lower interleukin-12P70 levels, longer fever and cough durations, and higher lactate dehydrogenase levels (all *P* < 0.05). Correlation analysis showed strong associations between white blood cells, platelets, and proinflammatory cytokines. ROC analysis of single parameters showed modest discrimination: for bacterial co-infections the best single marker was interferon-alpha with an AUC of 0.774 (95% CI 0.649–0.899), whereas for viral co-infections the best was lactate dehydrogenase with an AUC of 0.684 (95% CI 0.563–0.806). In contrast, machine learning models achieved higher performance, reaching an AUC of 0.868 (95% CI 0.772–0.964; SVM) for bacterial co-infections and 0.753 (95% CI 0.636–0.870; LightGBM) for viral co-infections.

**Conclusion:**

The combination of clinical data, laboratory parameters and cytokine profiles may improve the early diagnostic accuracy of MPP with bacterial or viral co-infections in some patients, and potentially guide optimised treatment strategies.

**Supplementary Information:**

The online version contains supplementary material available at 10.1186/s12887-026-06992-3.

## Background

*Mycoplasma pneumoniae* (MP) is widely recognised as the primary causative agent of community-acquired pneumonia, particularly among school-aged children, and is a significant contributor to the global burden of respiratory illnesses [1]. In recent years, notably in the last decade, there has been a discernible increase in severe and refractory *Mycoplasma pneumoniae* pneumonia (MPP) cases, particularly in regions such as China. These instances often exhibit increased severity and demonstrate resistance to established macrolide therapies [2–4]. Clinically, MPP presents with fever, persistent cough, sore throat, headaches, and rhinitis. Although these symptoms are typically manageable in milder forms, severe cases may evolve into critical complications such as pulmonary consolidation, atelectasis, lung abscesses, respiratory distress syndrome, and bronchiectasis. These complications can reduce respiratory efficiency and significantly diminish the quality of life of the affected children [5]. Additionally, severe MPP can initiate chronic airway diseases and obstructive pulmonary disorders, leading to prolonged impairment of lung function and restricted pulmonary development [6].

A significant factor contributing to the increased severity of MPP is its association with mixed infections. These co-infections cause clinical challenges by impairing ciliary function, fostering mucus accumulation, and diminishing immune response [7]. Such dynamics are believed to increase the risk of severe or refractory pneumonia, complicating early diagnosis and treatment because of atypical symptoms [8]. Cytokines play a crucial role in modulating the immune response to MP infection. Proinflammatory cytokines (IL-6, IL-10, IFN-γ, TNF-α, IL-18) regulate host immunity during Mycoplasma pneumoniae (MP) infection [9]. IL-17 A and IL-6 demonstrate distinct clinical correlations: IL-17 A associates with diffuse bronchiolitis and lobar lesions via mucus hypersecretion, while IL-6 correlates with lobar necrosis [10]. Refractory MP pneumonia (RMPP) exhibits altered macrophage chemokine profiles—elevated CXCL10 with reduced CCL3 and CCL11—relative to non-refractory cases [11]. In mixed infections, the cytokine-mediated inflammatory response is more pronounced. The interaction between MP and concurrent pathogens can precipitate an excessive release of proinflammatory cytokines, which further deteriorates respiratory function and amplifies clinical symptoms.

Despite the critical role of cytokines in exacerbating MPP associated with mixed infections, analyses of the cytokine profiles in these cases are lacking. This study explored the diagnostic potential of cytokine profiles in MPP complicated by bacterial or viral co-infections. Our ultimate objective was to refine the early detection methods and optimise treatment strategies, thereby improving the clinical outcomes of affected patients.

## Materials and methods

This retrospective study was conducted at a paediatric hospital in Fujian Province and included 145 children diagnosed with MPP between October 2023 and April 2024. All participants presented with clinical manifestations typical of MP infection, including fever and cough.

### Inclusion and exclusion criteria

The inclusion criteria were based on guidelines for the diagnosis and management of MPP in children. Patients were included if they met one or both of the following conditions, in addition to clinical and radiographic features [12]:

1. A single serum MP antibody titre ≥ 1:160 (measured by the particle agglutination method) or a fourfold or greater increase in paired serum MP antibody titres during the disease course.
2. Positive MP-DNA and RNA detection results.

The exclusion criteria were as follows.

1. Age over 16 years.
2. Readmission within 28 days after discharge.
3. History of asthma, tuberculosis, chronic malnutrition, aspiration pneumonia, immunodeficiency, cystic fibrosis, primary ciliary dyskinesia, or bronchopulmonary dysplasia.
4. Incomplete clinical data.

The study was approved by the Medical Ethics Committee of Fujian Children’s Hospital (approval no. 2024 ETKLRK10003) and was conducted in accordance with the ethical principles of the Declaration of Helsinki.

### Patient grouping

Patients were categorised into three groups based on the presence and type of co-infection.

1. MPP: Children diagnosed with MPP exclusively, with no other respiratory pathogens identified.
2. MPP with bacterial co-infection group (MPP_bacteria): Children diagnosed with MPP and concurrent bacterial infection.Bacterial co-infection was confirmed by isolating bacterial pathogens from sputum culture. To minimize the risk of misclassifying bacterial colonization as active infection, all cases were independently reviewed by two senior paediatric pulmonologists who were blinded to the cytokine results and to each other’s assessment. The evaluation considered clinical features (such as persistent high fever and pathogen-specific symptoms), radiological findings (e.g., progressive pulmonary consolidation), and laboratory results (including elevated white blood cell counts, neutrophil proportions, and inflammatory markers). Through this comprehensive assessment, asymptomatic carriers were excluded. No additional respiratory pathogens were detected in this group.
3. MPP with viral co-infection group (MPP_virus): children diagnosed with MPP and concurrent viral infection. Viral co-infection was confirmed by the detection of viruses using polymerase chain reaction, antigen tests, or serological methods, ensuring that no other types of respiratory pathogens were detected.

### Data collection

Demographic data, clinical symptoms, and laboratory test results were retrospectively collected from the medical records of the 145 patients. The evaluation parameters included age, sex, fever duration before admission, highest recorded body temperature, and cough duration before admission. Routine laboratory tests conducted within 24 h of admission included the following:

Haematological tests included white blood cell (WBC) count, neutrophil percentage (N%), lymphocyte percentage, platelet (PLT) count, C-reactive protein (CRP), lactate dehydrogenase (LDH), serum ferritin, D-dimer level, erythrocyte sedimentation rate, procalcitonin (PCT), and alanine aminotransferase.

### Cytokine analysis

Cytokine profiling included interleukin (IL)-1β, IL-2, IL-4, IL-5, IL-6, IL-8, IL-10, IL-12P70, IL-17 A, tumour necrosis factor (TNF)-α, interferon (IFN)-α, and IFN-γ. The concentrations of the target cytokines in serum were quantified using a multiplex bead-based immunofluorescence assay. All procedures were performed following the manufacturer’s protocol for the specific detection kit (Tianjin Botongsheng Biotechnology Co., Ltd., China; Registration No. 20222400202). Venous blood samples were collected in tubes containing inert separation gel and coagulants. Serum was separated by centrifugation at 1000–1200 g for 15 min and stored at -80 °C until analysis to avoid repeated freeze-thaw cycles. For the assay, 20 µL of each serum sample was mixed with 20 µL of antibody-coated capture microspheres and 20 µL of biotinylated detection antibody mixture in a dedicated flow tube. The reaction mixture was incubated at room temperature (23–27 °C) for 2 h in the dark with continuous shaking at 500 rpm. Subsequently, 20 µL of phycoerythrin-labeled streptavidin (SA-PE) was added, followed by an additional 30-minute incubation under the same conditions. The microspheres were then washed twice with 300 µL of 1× wash buffer via centrifugation at 500 g for 5 min. After discarding the supernatant, the pellets were resuspended in 150–300 µL of 1× wash buffer for data acquisition. Data were acquired using a BD FACSCanto II flow cytometer (BD Biosciences, USA). To ensure statistical robustness, a minimum of 100 microspheres was collected for each cytokine population. The final concentrations were calculated based on a 9-point calibration curve generated using recombinant protein standards provided in the kit.

### Statistical methods

Statistical analysis was performed using R software (version 4.4.1; R Foundation for Statistical Computing, Vienna, Austria). The normality of continuous variables was assessed using the Kolmogorov–Smirnov test. Normally distributed data were expressed as mean ± SD and compared using t-tests or ANOVA, while non-normally distributed data were presented as medians with interquartile ranges (IQR) and analysed using the Mann–Whitney U test or the Kruskal–Wallis test. For post-hoc pairwise comparisons following a significant Kruskal–Wallis test, Dunn’s test with Bonferroni correction was applied. Categorical variables were compared using the chi-square test or Fisher’s exact test.

Correlation analysis was performed using Pearson’s or Spearman’s coefficients, depending on data distribution. Network visualisations of feature correlations were generated using Cytoscape (version 3.4.0), with nodes representing variables and edges defined as correlations of |r| > 0.3 and *P* < 0.05; node size and edge thickness reflected statistical significance and correlation strength, respectively. Correlation matrices for network construction were computed separately within each infection group using Spearman’s rank correlation (pairwise complete observations). Edges were retained when |r| > 0.30 with *P* < 0.05.

The diagnostic efficiency of key parameters was evaluated using receiver operating characteristic (ROC) curves, with area under the curve (AUC), cutoff values, sensitivity, and specificity reported.

Machine learning models, including random forest (RF), Light Gradient Boosting Machine (LightGBM), and support vector machine (SVM), were applied to classify bacterial and viral co-infections. Feature selection was conducted using the Boruta algorithm, and feature importance was additionally assessed using RF and XGBoost. Model performance was evaluated with 5-fold cross-validation to avoid overfitting, and predictive ability was quantified by AUC with 95% confidence intervals. All numeric predictors were z-standardized within training folds; missing values (if any) were imputed by within-fold medians. Cross-validation was stratified by outcome, and hyperparameters were tuned via grid search within each training fold. AUC 95% confidence intervals were calculated using DeLong’s method, and ROC cutoffs were selected using the Youden index. For ROC analyses and model construction, comparisons were performed pairwise: (i) MPP_bacteria vs. MPP, and (ii) MPP_virus vs. MPP. The two co-infection groups were not combined into a single reference group, in order to address two independent clinical questions.

Data visualisation was performed using ggplot2 and pheatmap packages in R. Statistical significance was defined as *P* < 0.05, with Bonferroni correction for multiple comparisons where appropriate.

## Results

### Clinical characteristics and inflammatory indices

Among the 145 enrolled patients, 97, 19, and 29 were classified into the MPP group, MPP_bacteria group, and MPP_virus group, respectively. The demographic and clinical characteristics of the three groups are listed in Table 1.

**Table 1:** Comparison of clinical characteristics and inflammatory indices between MPP and MPP_bacteria and MPP and MPP_virus groups

Form	MPP	MPP_bacteria	MPP_virus	P_MB	P_MV
Age (years)	6.00 (4.00 ~ 8.00)	5.00 (4.00 ~ 6.50)	5.00 (4.00 ~ 6.00)	0.165	0.018
Sex (n)					
Male	43.00	8.00	12.00	1.000	0.946
Female	54.00	11.00	17.00		
Duration of fever before admission (days)	5.00 (4.00 ~ 6.00)	4.00 (2.50 ~ 5.00)	6.00 (5.00 ~ 9.00)	0.082	0.011
Peak temperature (℃)	39.30 (38.70 ~ 39.80)	39.00 (38.50 ~ 39.70)	39.60 (39.20 ~ 40.00)	0.242	0.051
Cough duration (days)	7.00 (5.00 ~ 10.00)	7.00 (6.50 ~ 14.00)	9.00 (6.00 ~ 13.00)	0.115	0.042
WBC (×10^9^/L)	7.50 (6.58 ~ 9.53)	11.70 (9.81 ~ 14.02)	8.63 (7.42 ~ 11.84)	0.001	0.099
N%	61.55 ± 12.37	59.54 ± 12.95	63.73 ± 12.56	0.539	0.414
L%	28.20 (21.50 ~ 39.80)	32.00 (23.30 ~ 35.95)	29.10 (21.90 ~ 31.80)	0.460	0.860
PLT (×10^9^/L)	287.00 (239.00 ~ 383.00)	407.00 (358.50 ~ 486.50)	320.00 (259.00 ~ 406.00)	0.002	0.364
CRP (mg/L)	7.18 (3.23 ~ 19.97)	7.53 (2.02 ~ 32.87)	6.00 (2.16 ~ 9.83)	0.835	0.235
LDH (U/L)	275.00 (245.00 ~ 322.00)	254.00 (232.00 ~ 276.00)	315.00 (263.00 ~ 351.00)	0.142	0.031
Serum ferritin (ng/mL)	164.20 (115.30 ~ 223.60)	155.10 (111.80 ~ 225.10)	180.00 (140.90 ~ 281.90)	0.893	0.161
D-dimer (ng/L)	0.44 (0.33 ~ 0.76)	0.50 (0.46 ~ 0.74)	0.53 (0.30 ~ 0.68)	0.526	0.681
ESR (mm/h)	43.33 ± 17.75	47.47 ± 25.60	46.66 ± 16.82	0.507	0.361
PCT (ng/mL)	0.10 (0.06 ~ 0.16)	0.13 (0.07 ~ 0.31)	0.09 (0.07 ~ 0.20)	0.396	0.805
ALT (U/L)	13.00 (9.00 ~ 19.00)	13.00 (8.50 ~ 15.00)	14.00 (10.00 ~ 19.00)	0.662	0.190

*MPP Mycoplasma pneumoniae* pneumonia, *P_MB* the p-value of MPP_bacteria, *P_MV*the *P* value of MPP_virus, *WBC* white blood cell, *N%* neutrophil percentage, *L%* lymphocyte percentage, *PLT* platelet, *CRP* C-reactive protein, *LDH* lactate dehydrogenase, *ESR* erythrocyte sedimentation rate, *ALT* alanine aminotransferase

*P* values are Bonferroni-corrected for multiple comparisons

The duration of fever before admission was significantly longer in the MPP_virus group (6.00 days, IQR: 5.00–9.00) compared to the MPP group (5.00 days, IQR: 4.00–6.00, *P =* 0.011), while the MPP_bacteria group showed no significant difference (4.00 days, IQR: 2.50–5.00, *P =* 0.082). Peak temperature exhibited a trend toward higher values in the MPP_virus group (39.60 °C, IQR: 39.20–40.00) compared to the MPP group (39.30 °C, IQR: 38.70–39.80) without reaching statistical significance (*P =* 0.051). Cough duration was significantly longer in the MPP_virus group (9.00 days, IQR: 6.00–13.00) compared to the MPP group (7.00 days, IQR: 5.00–10.00, *P =* 0.042).

Laboratory examination revealed distinct patterns among the groups. The MPP_bacteria group demonstrated significantly elevated WBC count (11.70 × 10^9^/L, IQR: 9.81–14.02) compared to the MPP group (7.50 × 10^9^/L, IQR: 6.58–9.53, *P =* 0.001). PLT counts were also notably higher in the MPP_bacteria group (407.00 × 10^9^/L, IQR: 358.50–486.50) versus the MPP group (287.00 × 10^9^/L, IQR: 239.00–383.00, *P =* 0.002). Neutrophil and lymphocyte percentages were not significantly different among the groups (*P* > 0.05). LDH levels were significantly elevated in the MPP_virus group (315.00 U/L, IQR: 263.00–351.00) compared to the MPP group (275.00 U/L, IQR: 245.00–322.00, *P =* 0.031) (Table 1).

### Distribution of co-infecting pathogens

Of 145 MPP cases, bacterial co-infection occurred in 19 (13.1%)—mainly S. pneumoniae (9) and H. influenzae (8), with B. pertussis (2). Viral co-infection occurred in 29 (36 detections in total): influenza (13), rhinovirus (8), adenovirus (7), human metapneumovirus (4), parainfluenza (2), human coronavirus (1), and RSV (1) (Table 2).

**Table 2:** Distribution of co-infecting pathogens in MPP

A. Bacterial co-infections			
Pathogen	*n* (patients)	% within bacterial co-infections	% of total cohort
Streptococcus pneumoniae	9	47.4	6.2
Haemophilus influenzae	8	42.1	5.5
Bordetella pertussis	2	10.5	1.4
Total	19	100.0	13.1

B. Viral co-infections			
Pathogen	*n* (detections)*	% within viral co-infections	% of total cohort
Influenza virus	13	44.8	9.0
Rhinovirus	8	27.6	5.5
Adenovirus	7	24.1	4.8
Human metapneumovirus	4	13.8	2.8
Parainfluenza virus	2	6.9	1.4
Human coronavirus	1	3.4	0.7
Respiratory syncytial virus	1	3.4	0.7
Total detections*	36		

*Abbreviations*: *MPP Mycoplasma pneumoniae* pneumonia, *RSV* respiratory syncytial virus

*For viral co-infections, counts reflect detections, because one patient may have ≥1 virus detected. Totals therefore exceed the number of viral-coinfected patients (*n*=29). Percentages for viral co-infections are calculated per viral-coinfected patients (*n* = 29)

### Cytokine expression profiles

Descriptive cytokine distributions are summarized (Table 3). Inferential findings are presented below in the group-wise comparison sections.

**Table 3:** Comparison of cytokine expression levels between MPP and MPP_bacteria and MPP and MPP_virus groups

Form (pg/mL)	MPP	MPP_bacteria	MPP_virus	P_MB	P_MV
IFN-α	2.36(1.41 ~ 4.82)	4.67(3.24 ~ 12.01)	3.68(1.62 ~ 6.75)	0.006	0.114
IFN-γ	1.52(0.79 ~ 3.06)	1.35(0.90 ~ 2.89)	1.29(0.82 ~ 2.35)	0.817	0.500
IL-10	5.43(3.23 ~ 7.53)	3.98(2.93 ~ 8.97)	4.67(3.67 ~ 8.18)	0.908	0.481
IL-12P70	1.20(0.00 ~ 2.34)	1.83(0.00 ~ 2.06)	0.00(0.00 ~ 1.25)	0.850	0.040
IL-17 A	6.71(3.51 ~ 11.46)	6.87(2.94 ~ 8.95)	7.21(3.51 ~ 12.58)	0.530	0.741
IL-1β	0.92(0.27 ~ 1.87)	2.24(0.61 ~ 4.48)	0.71(0.42 ~ 1.53)	0.023	0.783
IL-2	0.58(0.17 ~ 0.99)	1.31(0.32 ~ 1.53)	0.53(0.22 ~ 1.10)	0.056	0.972
IL-4	0.83(0.00 ~ 1.76)	1.16(0.11 ~ 2.17)	0.99(0.42 ~ 1.77)	0.226	0.183
IL-5	0.50(0.16 ~ 0.73)	0.57(0.31 ~ 0.79)	0.31(0.15 ~ 0.48)	0.490	0.174
IL-6	9.60(4.43 ~ 18.35)	18.84(10.73 ~ 26.79)	9.83(3.69 ~ 19.74)	0.038	0.998
IL-8	12.11(8.50 ~ 17.29)	13.79(8.29 ~ 16.84)	11.65(8.63 ~ 24.32)	0.671	0.284
TNF-α	1.39(0.72 ~ 2.21)	2.48(1.19 ~ 4.14)	1.03(0.45 ~ 2.52)	0.026	0.639

*MPP* *Mycoplasma pneumoniae* pneumonia, *P_MB* p-value of MPP_bacteria, *P_MV* *P* value of MPP_virus, *IFN* interferon, *IL* interleukin, *TNF* tumour necrosis factor

*P* values are Bonferroni-corrected for multiple comparisons

### Feature correlation network analysis

Correlation network analysis revealed complex interactions between clinical parameters and inflammatory markers. WBC and PLT showed broad connectivity with multiple cytokines, particularly pro-inflammatory markers such as IL-6 and TNF-α; LDH was embedded within the cytokine cluster (Supplementary Figure S1A–S1B**).** Statistical analyses suggested a two-module structure separating clinical parameters from cytokine levels (Supplementary Figure S1C). Inferential findings are presented below in the group-wise comparison sections.

### Machine learning analysis

**Fig. 1:**
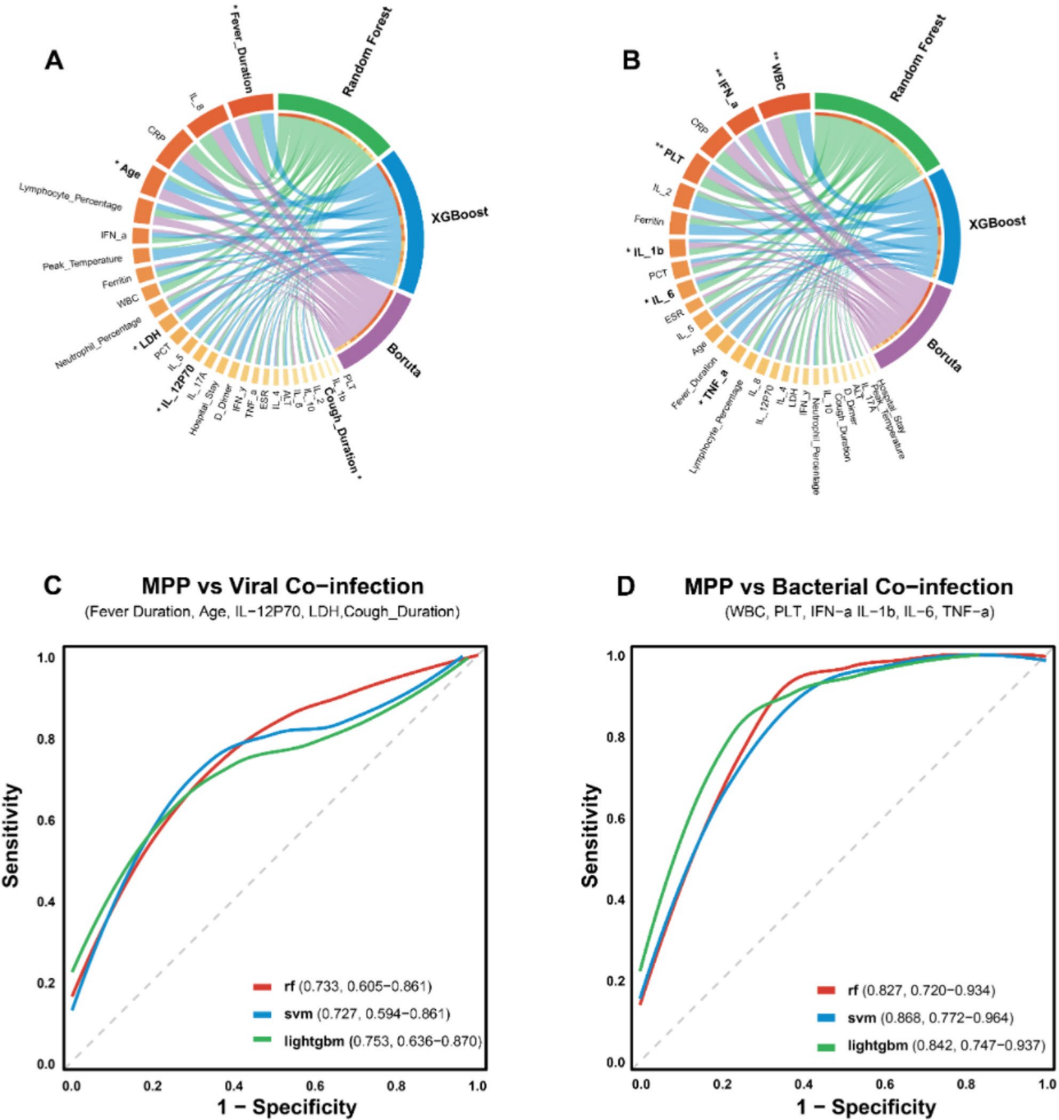
Machine learning analysis for co-infection classification. **a**, **b** Feature importance analysis by three algorithms. **c**, **d** Performance comparison of three machine learning models (RF, SVM, and LightGBM) in predicting viral and bacterial co-infections. RF, random forest; SVM, support vector machine; LightGBM, Light Gradient Boosting Machine; PLT, platelet; IL, interleukin; ALT, alanine aminotransferase; ESR, erythrocyte sedimentation rate; TNF, tumour necrosis factor; IFN, interferon; PCT, procalcitonin; LDH, lactate dehydrogenase; WBC, white blood cell; CRP, C-reactive protein; MPP, *Mycoplasma pneumoniae* pneumonia

Feature selection methods (random forest, XGBoost, and Boruta algorithms) identified five key parameters (duration of fever before admission, age, IL-12P70, LDH, and cough duration) for distinguishing MPP from viral co-infection, and six parameters (WBC, PLT, IFN-α, IL-1β, IL-6, and TNF-α) for bacterial co-infection classification (Fig. 1A, B). For viral co-infection detection, LightGBM achieved the highest AUC of 0.753 (95% confidence interval [CI]: 0.636–0.870), followed by random forest (AUC=0.733), and SVM (AUC=0.727, Fig. 1C). For bacterial co-infection classification, SVM demonstrated the best performance, with an AUC of 0.868 (95% CI: 0.772–0.964), followed by LightGBM (AUC=0.842), and random forest (AUC=0.827, Fig. 1D) (Supplementary Table S1). 

### Parameters in MPP versus bacteria co-infection groups

**Fig. 2:**
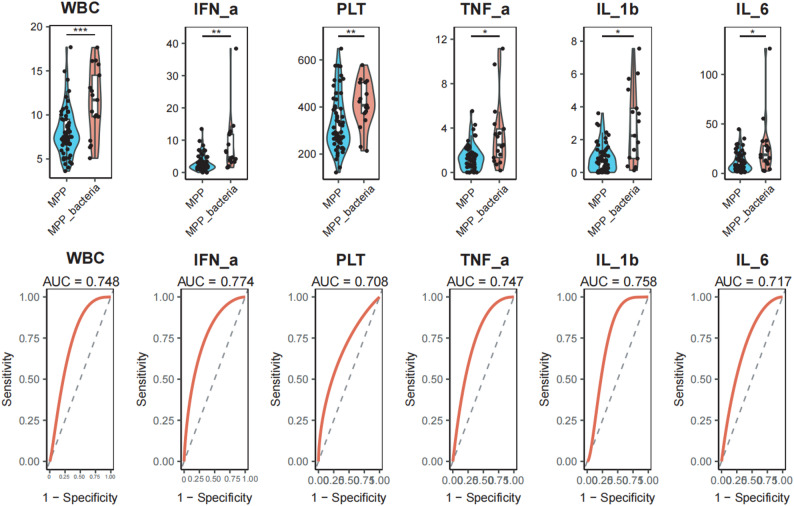
MPP vs. bacterial co-infection comparison. Violin plots and ROC curves of six key parameters (WBC count, IFN-α, PLT count, TNF-α, IL-1β, and IL-6) demonstrating their diagnostic value in distinguishing bacterial co-infection. WBC, white blood cell; IFN, interferon; PLT, platelet; TNF, tumour necrosis factor; IL, interleukin; MPP, *Mycoplasma pneumoniae* pneumonia; AUC, area under the curve

Six key parameters were significantly different between the MPP and MPP_bacteria groups (Table 4; Fig. 2). The MPP_bacteria group demonstrated significantly higher WBC counts (*P* < 0.01); IFN-α and PLT levels (*P* < 0.01); and TNF-α, IL-1β, and IL-6 levels (*P* < 0.05). ROC analysis revealed that IFN-α had the highest diagnostic value (AUC = 0.774), followed by IL-1β (AUC = 0.758), WBC (AUC = 0.748), TNF-α (AUC = 0.747), IL-6 (AUC = 0.717), and PLT counts (AUC = 0.708).

**Table 4:** Performance comparison between MPP vs. co-infection groups

Comparison	Variable	AUC (95% CI)	Cutoff	Sensitivity	Specificity
MPP vs. virus	Fever Duration	0.656 (0.512–0.799)	8.5	0.948	0.346
	Age	0.601 (0.481–0.721)	7.5	0.276	0.962
	LDH	0.684 (0.563–0.806)	278.5	0.603	0.692
	Cough Duration	0.682 (0.554–0.809)	10.5	0.879	0.385
	IL-12P70	0.652 (0.522–0.782)	0.115	0.707	0.615
MPP vs. bacteria	WBC	0.748 (0.593–0.904)	9.665	0.776	0.765
	IFN-α	0.774 (0.649–0.899)	3.125	0.69	0.824
	PLT	0.708 (0.571–0.845)	343.5	0.638	0.824
	TNF-α	0.747 (0.602–0.892)	2.32	0.862	0.588
	IL-1β	0.758 (0.611–0.906)	3.04	0.983	0.471
	IL-6	0.717 (0.568–0.866)	9.1	0.586	0.824

*MPP* *Mycoplasma pneumoniae* pneumonia, *AUC* area under the curve, *CI* confidence interval, *LDH* lactate dehydrogenase, *IL* interleukin, *WBC* white blood cell, *IFN* interferon, *PLT* platelet, *TNF* tumour necrosis factor

### Parameters in MPP versus viral co-infection groups

**Fig. 3:**
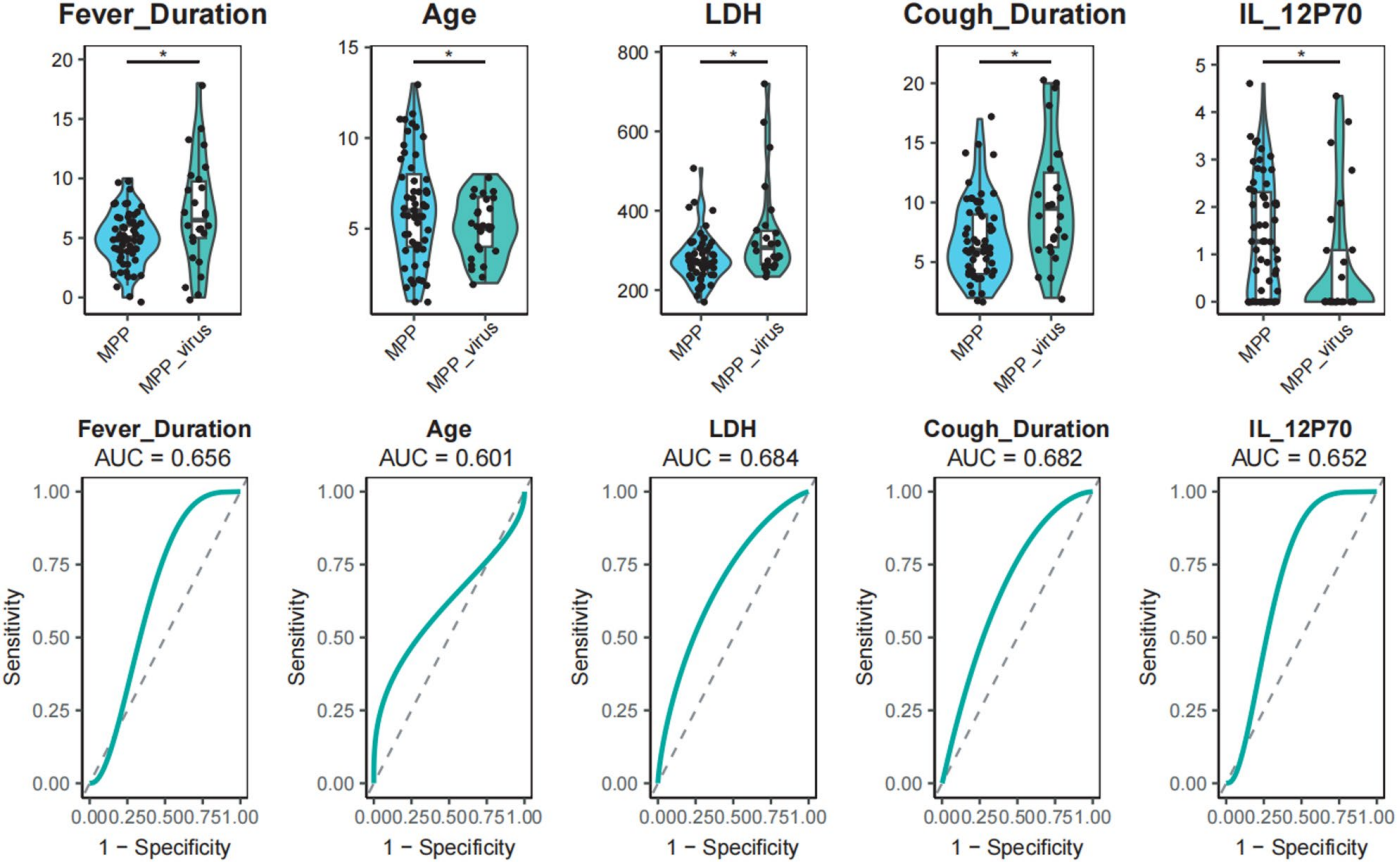
MPP vs. viral co-infection comparison. Violin plots and ROC curves of five key parameters (fever duration, age, LDH, cough duration, IL-12P70) demonstrating their diagnostic value in distinguishing viral co-infection. LDH, lactate dehydrogenase; IL, interleukin; MPP, *Mycoplasma pneumoniae* pneumonia; AUC, area under the curve

Five key parameters showed significant differences between the MPP and MPP_virus groups (*P <* 0.05; Table 4; Fig. 3). The MPP_virus group exhibited a longer fever duration with a broader upper-range distribution, younger age, elevated LDH levels with a wider upper-range spread, prolonged cough duration, and lower IL-12P70 levels with a concentrated lower-range distribution. ROC analysis revealed moderate diagnostic capabilities, with LDH exhibiting the highest AUC (0.684), followed by cough duration (AUC = 0.682), fever duration (AUC = 0.656), IL-12P70 levels (AUC = 0.652), and age (AUC = 0.601).

### Subgroup analysis

Subgroup analyses were performed based on the co-infecting pathogens. In the bacterial co-infection subgroups, both *Streptococcus*-MPP and *Haemophilus influenzae*-MPP groups exhibited significantly higher levels of WBC, PLT, and IFN-α than MPP alone (*P =* 0.013, 0.0268, and 0.0173, respectively; Supplementary Figure S2). IL-6 levels were also significantly elevated (*P =* 0.0273), whereas differences in IL-1β and TNF-α levels were not statistically significant (*P =* 0.0955 and 0.1639, respectively; Supplementary Figure S2). Among the viral co-infection subgroups, IL-12P70 levels were significantly lower in all viral subgroups than in MPP alone, particularly in the adenovirus (ADV)-MPP group (*P =* 0.0104, Supplementary Figure S2). The ADV-MPP group had the highest LDH levels (*P =* 0.0414) and longest cough duration (*P =* 0.0087), followed by the influenza virus- and rhinovirus-MPP groups (Supplementary Figure S2). The fever duration and age distribution did not differ significantly between the viral subgroups (*P =* 0.0689 and 0.1978, respectively; Supplementary Figure S2).

## Discussion

In recent years, the incidence of MPP in children has significantly increased, presenting substantial challenges for paediatric healthcare systems [13]. MPP may present as a standalone infection or coincide with bacterial or viral pathogens, complicating the clinical picture and delaying targeted antimicrobial or antiviral treatment. These complexities not only impact individual patient outcomes but also exert considerable strain on public health infrastructure. Consequently, prompt identification and effective management of mixed infections are critical for the development of targeted therapies and for enhancing the overall patient prognosis.

Children with MPP and concurrent bacterial co-infections exhibited higher WBC and PLT counts than those with MPP alone. This result is consistent with previous studies suggesting that bacterial infections elicit inflammatory responses, thereby activating host defense mechanisms, such as innate immune cell mobilization, cytokine-mediated inflammation, and neutrophil extracellular trap formation [14]. Elevated WBC counts are a hallmark of bacterial infections, reflecting enhanced bone marrow activity and mobilisation of peripheral granulocytes [15]. Similarly, increased PLT counts may result from the activation of endothelial cells and coagulation pathways, which are frequently involved in bacterial infections. In addition to their well-established role in haemostasis, PLTs play a role in immune responses by expressing immune receptors and releasing antimicrobial peptides [16]. These observations underscore the complex adaptive mechanisms used by the body to combat bacterial infections.

Interestingly, standard inflammatory markers, such as CRP and PCT, did not effectively differentiate between isolated MPP infections and bacterial co-infections. Although CRP levels may escalate significantly owing to severe tissue damage, particularly in cases of MPP with extensive lung involvement, such increases are not unique to bacterial infections [17]. Moreover, the existing literature suggests that elevated PCT levels can be observed in MPP cases and may act as independent risk factors for prolonged fever and longer hospital stay [18]. Hence, CRP and PCT may not be reliable indicators for identifying co-infections in MPP cases, highlighting the necessity for alternative diagnostic markers.

Our analysis demonstrated significant differences in the duration of fever before admission, cough duration, and LDH levels between children with viral co-infections and those with isolated MPP infections. These findings support the hypothesis that viral infections exacerbate conditions by impairing host immune responses, damaging respiratory epithelial barriers, and promoting secondary infections [19]. Furthermore, elevated LDH levels, which indicate tissue damage and cellular turnover, have been associated with severe pneumonia in patients with viral co-infections at admission [20]. These observations underscore the critical need for the early recognition and proactive management of viral co-infections in MPP to mitigate disease severity and enhance patient outcomes.

Cytokine profiling revealed that patients with MPP and bacterial co-infections exhibited significantly elevated levels of IFN-α, IL-1β, IL-6, and TNF-α than those with isolated MPP infections. Additionally, IL-12P70 levels were significantly lower in patients with viral co-infections. These increases in cytokine levels were consistent with enhanced immune and inflammatory responses. IFN-α is crucial for the initiation of adaptive immunity [21]. Peignier et al. demonstrated the complex role of IFN-α in modulating susceptibility to bacterial infections, underscoring the differences in responses to bacterial and viral pathogens [22]. IL-1β facilitates the recruitment and activation of immune cells, which correlates strongly with the severity of infection [23]. IL-6 plays a significant role in the acute phase response, promoting T cell proliferation and B cell differentiation, and is an early indicator of systemic infections, such as sepsis [24]. TNF-α is integral in mediating inflammatory responses to bacterial pathogens, aiding in immune cell activation, and regulating apoptosis [25]. Interestingly, while our results showed reduced IL-12P70 levels in viral co-infections, previous studies reported elevated IL-12P70 in similar settings [26]. This discrepancy may reflect differences in patient cohorts, stages of infection, or the dynamic regulation of IL-12P70 during viral immune responses, highlighting the need for further investigation.

Conversely, several studies have reported increased IL-6 levels in viral infections, including COVID-19, underscoring its potential as a biomarker of disease severity [27]. Furthermore, IL-12 has been implicated in immune responses to bacterial infections, reflecting the intricate interactions between cytokines in different infections [28]. These variations may originate from differences in pathogen types, host immune responses, or methodologies employed in these studies. Our findings emphasise the importance of contextualising cytokine profiles within a specific infectious milieu when interpreting cytokine levels.

By incorporating clinical data, routine laboratory tests, and cytokine measurements, the integrated approach may improve identification of MPP cases complicated by bacterial or viral co-infections. This integrated approach may provide insights into the early diagnosis and management of mixed infections in patients with MPP. In support of this, previous research has endorsed the use of combined cytokine assessments to enhance diagnostic precision, particularly in MPP cases with adenovirus co-infection [29]. Although leveraging multiple biomarkers yields considerable diagnostic advantages, it also raises concerns regarding increased healthcare expenditures and resource allocation. Future research should perform cost-benefit analyses to determine the most efficient combinations of diagnostic tests or develop more accessible and cost-effective methods for cytokine detection.

By investigating the clinical features of MPP co-infections, we identified several significantly different characteristic factors and conducted detailed classification analyses. *Streptococcus pneumoniae* and *Haemophilus influenzae* were the predominant bacteria in the cases of bacterial co-infection, with distinct differences in characteristic factors across various co-infection groups. Specifically, patients co-infected with *H. influenzae* had significant variations in WBC count, IFN-α, and IL-6 compared to those co-infected with MPP alone. Additionally, the PLT count differed markedly between the MPP-alone group and patients coinfected with *S. pneumoniae*. Rhinovirus, influenza virus, and adenovirus were commonly observed in MPP patients. In this study, patients with MPP co-infected with adenovirus showed different IL-12P70 levels and longer cough duration compared with those with MPP alone. These findings may reflect differences in pathogenic mechanisms among pathogens co-infecting with MPP, which can lead to distinct clinical manifestations and inflammatory responses. Previous studies have reported a close association between adenovirus co-infection and the development of refractory Mycoplasma pneumoniae pneumonia (RMPP) [30]. Currently, research exploring the differences among various pathogen co-infections in MPP is limited, particularly concerning bacterial co-infections. Therefore, further in-depth studies on the pathogenic mechanisms of MPP co-infection with different pathogens are crucial for developing effective treatment strategies and improving patient prognosis.

From a clinical perspective, given the delay in pathogen detection results, the findings of this study support the feasibility of ‘precision treatment’ even before microbiological results are available: (i) When a high probability of bacterial co-infection is indicated, it is reasonable to combine macrolide therapy for Mycoplasma pneumoniae (MP) with other appropriate antibiotics to cover common bacterial pathogens, thereby mitigating the risk of disease progression [31]. (ii) Conversely, when cytokine-based assessments suggest a low likelihood of bacterial co-infection, clinicians may consider withholding or de-escalating broad-spectrum antibiotic use, thereby reducing unnecessary drug exposure and adverse reactions [32]. (iii) Previous studies have demonstrated that viral co-infections are associated with aggravated disease progression and an increased risk of RMPP or severe pneumonia [20]. In cases where viral co-infection is suspected, intensified monitoring and timely supportive care are warranted. Furthermore, when clear indications are present, antiviral or immunomodulatory therapies may be considered.

This study had certain limitations. First, its retrospective design may have introduced selection and information bias, while the relatively small sample size may have limited the generalisability of our findings. Second, there was no external validation cohort, potentially inflating performance estimates and limiting generalisability. Moreover, the research was conducted within specific geographical and temporal contexts, which may not fully represent the diversity of paediatric populations or consider regional differences in pathogen prevalence. Future studies should employ prospective multicentre approaches involving larger sample sizes to validate and enhance the applicability of these findings. In particular, multicentre, large-sample prospective cohorts could provide an opportunity to conduct dedicated analyses of patients with concomitant viral–bacterial co-infections. In addition, implementing longitudinal designs could also be beneficial, as they would enable the assessment of outcomes over time, thereby providing more solid evidence to support clinical guidelines. Future prospective studies should adopt more stringent diagnostic criteria, incorporating more objective and reliable microbiological standards, such as quantitative bacterial culture thresholds, to further validate and refine our findings. Finally, although our study adopted binary classification to address two clinically distinct questions, future research with larger sample sizes should explore multi-class models to provide a more comprehensive diagnostic framework.

In conclusion, markers such as WBC count, PLT count, IFN-α, IL-1β, IL-6, and TNF-α may serve as useful indicators of bacterial co-infection in patients with MPP, whereas age, fever duration, cough duration, LDH, and IL-12P70 levels may help identify viral co-infections. Integrating these markers may potentially enhance the diagnostic approach for MPP with bacterial or viral co-infections and provide supportive information for clinical decision-making.

## Supplementary Information

Supplementary Material 1: Supplementary Figure S1. Correlation networks and significance heatmap. (a, b) Correlation networks between parameters in different infection groups. (c) Significance heatmap of parameters categorised by clinical characteristics and cytokines (*P* < 0.05, *P* < 0.01, *P* < 0.001). Supplementary Figure S2. Subgroup analysis by pathogens. Violin plots showing differences in key parameters among bacterial subgroups (SP-MPP, HI-MPP) and viral subgroups (RV-MPP, IV-MPP, ADV-MPP) compared with MPP alone. Supplementary Table S1. Detailed machine learning metrics for each model (AUC with 95% CI, sensitivity, specificity, and cutoff values). Abbreviations: MPP, *Mycoplasma pneumoniae* pneumonia; SP, *Streptococcus pneumoniae*; HI, *Haemophilus influenzae*; RV, rhinovirus; IV, influenza virus; ADV, adenovirus; WBC, white blood cell; PLT, platelet; LDH, lactate dehydrogenase; IFN, interferon; IL, interleukin; TNF, tumour necrosis factor; AUC, area under the curve; CI, confidence interval.

## Data Availability

Data supporting the findings of this study are available from Qiuyu Tang upon reasonable request.

## Abbreviations

ADV: Adenovirus
AUC: Area under the curve
CI: Confidence interval
CRP: C-reactive protein
IFN: Interferon
IQR: Interquartile range
IL: Interleukin
LDH: Lactate dehydrogenase
LightGBM: Light Gradient Boosting Machine
TNF: Tumour necrosis factor
MP: Mycoplasma pneumoniae
MPP: Mycoplasma pneumoniae pneumonia
PLT: Platelet
PCT: Procalcitonin
ROC: Receiver operating characteristic
SVM: Support vector machine
WBC: White blood cell
RMPP: Refractory Mycoplasma pneumoniae pneumonia

## References

[1] Fei F, Jun L, Qianyuan Y, Fei J. Clinical characteristics and serum inflammatory markers of community-acquired mycoplasma pneumonia in children. Clin Respir J. 2023;17:607–17. 10.1111/crj.136202. Conroy G. 37142438 10.1111/crj.13620 PMC10363789

[2] What’s behind China’s mysterious wave of childhood pneumonia? Nature. Published online November 27, 2023. 10.1038/d41586-023-03732-w. 10.1038/d41586-023-03732-w 38012356

[3] Looi MK, China. Rising cases of respiratory disease and pneumonia spark WHO concern. BMJ. 2023;383:2770. 10.1136/bmj.p2770. 37996101 10.1136/bmj.p2770

[4] Yen MH, Yan DC, Wang CJ, Tsao KC, Huang YC, Cheng SW, et al. The clinical significance of and the factors associated with macrolide resistance and poor macrolide response in pediatric Mycoplasma pneumoniae infection: A retrospective study. J Microbiol Immunol Infect. 2023;56:634–40. 10.1016/j.jmii.2023.01.010. 36737359 10.1016/j.jmii.2023.01.010

[5] Li QL, Wu YY, Sun HM, Gu WJ, Zhang XX, Wang MJ, et al. The role of miR-29c/B7-H3/Th17 axis in children with Mycoplasma pneumoniae pneumonia. Ital J Pediatr. 2019;45:61. 10.1186/s13052-019-0655-5. 31088519 10.1186/s13052-019-0655-5 PMC6518711

[6] Liu J, He R, Zhang X, Zhao F, Liu L, Wang H, et al. Clinical features and early corticosteroid treatment outcome of pediatric mycoplasma pneumoniae pneumonia. Front Cell Infect Microbiol. 2023;13:1135228. 10.3389/fcimb.2023.1135228. 37082710 10.3389/fcimb.2023.1135228 PMC10110948

[7] Kutty PK, Jain S, Taylor TH, Bramley AM, Diaz MH, Ampofo K, et al. Mycoplasma pneumoniae among children hospitalized with community-acquired pneumonia. Clin Infect Dis. 2019;68:5–12. 10.1093/cid/ciy419. 29788037 10.1093/cid/ciy419 PMC6552676

[8] Zhang X, Chen Z, Gu W, Ji W, Wang Y, Hao C, et al. Viral and bacterial coinfection in hospitalised children with refractory Mycoplasma pneumoniae pneumonia. Epidemiol Infect. 2018;146:1384–8. 10.1017/S0950268818000778. 29970200 10.1017/S0950268818000778 PMC9133674

[9] Tong L, Huang S, Zheng C, Zhang Y, Chen Z. Refractory Mycoplasma pneumoniae pneumonia in children: early recognition and management. J Clin Med. 2022;11:2824. 10.3390/jcm11102824. 35628949 10.3390/jcm11102824 PMC9144103

[10] Wang H, Zhang Y, Zhao C, Peng Y, Song W, Xu W, et al. Serum IL-17A and IL-6 in paediatric Mycoplasma pneumoniae pneumonia: implications for different endotypes. Emerg Microbes Infect. 2024;13:2324078. 10.1080/22221751.2024.2324078. 38407218 10.1080/22221751.2024.2324078 PMC10997354

[11] Lee YC, Chang CH, Lee WJ, Liu TY, Tsai CM, Tsai TA, et al. Altered chemokine profile in refractory mycoplasma pneumoniae pneumonia infected children. J Microbiol Immunol Infect. 2021;54:673–9. 10.1016/j.jmii.2020.03.030. 32299786 10.1016/j.jmii.2020.03.030

[12] Zhao S, Chen Z, Liu H, Zhao D, Hong J, Lu Q. Key interpretations of the National Health Commission’s guidelines for diagnosis and treatment of Mycoplasma pneumoniae pneumonia in children (2023 Edition). J Clin Pediatr. 2023;41:224–8. 10.12372/jcp.2023.22e0475.

[13] Parums DV, Editorial. Outbreaks of post-pandemic childhood pneumonia and the re-emergence of endemic respiratory infections. Med Sci Monit Int Med J Exp Clin Res. 2023;29:e943312. 10.12659/MSM.943312. 10.12659/MSM.943312 PMC10702145 38037346

[14] Lonsdale DO, Shah RV, Lipman J. Infection, sepsis and the inflammatory response: mechanisms and therapy. Front Med. 2023;7:588863. 10.3389/fmed.2020.588863. 10.3389/fmed.2020.588863 PMC7738462 33344475

[15] Honda T, Uehara T, Matsumoto G, Arai S, Sugano M. Neutrophil left shift and white blood cell count as markers of bacterial infection. Clin Chim Acta. 2016;457:46–53. 10.1016/j.cca.2016.03.017. 27034055 10.1016/j.cca.2016.03.017

[16] Rondina MT, Garraud O, Schwertz H. Platelets and bacterial infections. In: Gresele P, Kleiman NS, Lopez JA, Page CP, editors. Platelets in Thrombotic and Non-Thrombotic Disorders: Pathophysiology, Pharmacology and Therapeutics: An Update. New York: Springer International Publishing; 2017. pp. 1071–84.

[17] Wang J, Mao J, Chen G, Huang Y, Zhou J, Gao C, et al. Evaluation on blood coagulation and C-reactive protein level among children with mycoplasma pneumoniae pneumonia by different chest imaging findings. Med (Baltim). 2021;100:e23926. 10.1097/MD.0000000000023926. 10.1097/MD.0000000000023926 PMC7837868 33545964

[18] Jeong JE, Soh JE, Kwak JH, Jung HL, Shim JW, Kim DS, et al. Increased procalcitonin level is a risk factor for prolonged fever in children with Mycoplasma pneumonia. Korean J Pediatr. 2018;61:258–63. 10.3345/kjp.2018.61.8.258. 30130952 10.3345/kjp.2018.61.8.258 PMC6107399

[19] Denney L, Ho LP. The role of respiratory epithelium in host defence against influenza virus infection. Biomed J. 2018;41:218–33. 10.1016/j.bj.2018.08.004. 30348265 10.1016/j.bj.2018.08.004 PMC6197993

[20] Choo S, Lee YY, Lee E. Clinical significance of respiratory virus coinfection in children with Mycoplasma pneumoniae pneumonia. BMC Pulm Med. 2022;22:212. 10.1186/s12890-022-02005-y. 35637540 10.1186/s12890-022-02005-y PMC9150047

[21] Gharamti A, Samara O, Monzon A, Scherger S, DeSanto K, Sillau S, et al. Association between cytokine levels, sepsis severity and clinical outcomes in sepsis: a quantitative systematic review protocol. BMJ Open. 2021;11:e048476. 10.1136/bmjopen-2020-048476. 34373304 10.1136/bmjopen-2020-048476 PMC8354287

[22] Peignier A, Parker D. Impact of type I interferons on susceptibility to bacterial pathogens. Trends Microbiol. 2021;29:823–35. 10.1016/j.tim.2021.01.007. 33546974 10.1016/j.tim.2021.01.007 PMC8326292

[23] Volk CF, Burgdorf S, Edwardson G, Nizet V, Sakoulas G, Rose WE. Interleukin (IL)-1β and IL-10 host responses in patients with Staphylococcus aureus bacteremia determined by antimicrobial therapy. Clin Infect Dis. 2020;70:2634–40. 10.1093/cid/ciz686. 31365924 10.1093/cid/ciz686 PMC7286365

[24] Srisangthong P, Wongsa A, Kittiworawitkul P, Wattanathum A. Early IL-6 response in sepsis is correlated with mortality and severity score. Crit Care. 2013;17:P34. 10.1186/cc11972.

[25] Kassasseya C, Torsin LI, Musset C, Benhamou M, Chaudry IH, Cavaillon JM, et al. Divergent effects of tumor necrosis factor (TNF) in sepsis: a meta-analysis of experimental studies. Crit Care. 2024;28:293. 10.1186/s13054-024-05057-0. 39227889 10.1186/s13054-024-05057-0 PMC11373197

[26] Hamza T, Barnett JB, Li B. Interleukin 12 a key immunoregulatory cytokine in infection applications. Int J Mol Sci. 2010;11:789–806. 10.3390/ijms11030789. 20479986 10.3390/ijms11030789 PMC2869233

[27] Nikkhoo B, Mohammadi M, Hasani S, Sigari N, Borhani A, Ramezani C, et al. Elevated interleukin (IL)-6 as a predictor of disease severity among Covid-19 patients: a prospective cohort study. BMC Infect Dis. 2023;23:311. 10.1186/s12879-023-08294-w. 37161412 10.1186/s12879-023-08294-w PMC10169099

[28] Schurich A, Raine C, Morris V, Ciurtin C. The role of IL-12/23 in T cell–related chronic inflammation: implications of immunodeficiency and therapeutic blockade. Rheumatology. 2018;57:246–54. 10.1093/rheumatology/kex186. 28541488 10.1093/rheumatology/kex186

[29] Yi X, Jia W, Li W, Jia C, Song C. Diagnostic value of cytokines in severe childhood Mycoplasma pneumoniae pneumonia combined with Adenovirus infection. Ital J Pediatr. 2024;50:92. 10.1186/s13052-024-01661-6. 38715105 10.1186/s13052-024-01661-6 PMC11077701

[30] Zhou Y, Wang J, Chen W, Shen N, Tao Y, Zhao R, et al. Impact of viral coinfection and macrolide-resistant mycoplasma infection in children with refractory Mycoplasma pneumoniae pneumonia. BMC Infect Dis. 2020;20:633. 10.1186/s12879-020-05356-1. 32847534 10.1186/s12879-020-05356-1 PMC7447613

[31] Mathur S, Fuchs A, Bielicki J, Van Den Anker J, Sharland M. Antibiotic use for community-acquired pneumonia in neonates and children: WHO evidence review. Paediatr Int Child Health. 2018;38(sup1):S66–75. 10.1080/20469047.2017.1409455. 29790844 10.1080/20469047.2017.1409455 PMC6176769

[32] Song JU, Lee J. The impact of antimicrobial de-escalation therapy in culture-negative pneumonia: a systematic review and meta-analysis. Korean J Intern Med. 2023;38(5):704–13. 10.3904/kjim.2023.115. 37586813 10.3904/kjim.2023.115 PMC10493446

