# Supplementary material for: Diagnostic value of cytokine detection in children with Mycoplasma pneumoniae pneumonia complicated by bacterial or viral co-infections
Source: BMC Pediatr. 2026 May 14;26:629. doi: 10.1186/s12887-026-06992-3 (PMC13344005; doi:10.1186/s12887-026-06992-3)
Supplement: Supplementary file 1 — Supplementary Material 1: Supplementary Figure S1. Correlation networks and significance heatmap. (a, b) Correlation networks between parameters in different infection groups. (c) Significance heatmap of parameters categorised by clinical characteristics and cytokines (P < 0.05, P < 0.01, P < 0.001). Supplementary Figure S2. Subgroup analysis by pathogens. Violin plots showing differences in key parameters among bacterial subgroups (SP-MPP, HI-MPP) and viral subgroups (RV-MPP, IV-MPP, ADV-MPP) compared with MPP alone. Supplementary Table S1. Detailed machine learning metrics for each model (AUC with 95% CI, sensitivity, specificity, and cutoff values). Abbreviations: MPP, Mycoplasma pneumoniae pneumonia; SP, Streptococcus pneumoniae; HI, Haemophilus influenzae; RV, rhinovirus; IV, influenza virus; ADV, adenovirus; WBC, white blood cell; PLT, platelet; LDH, lactate dehydrogenase; IFN, interferon; IL, interleukin; TNF, tumour necrosis factor; AUC, area under the curve; CI, confidence interval. [file 12887_2026_6992_MOESM1_ESM.docx]

| 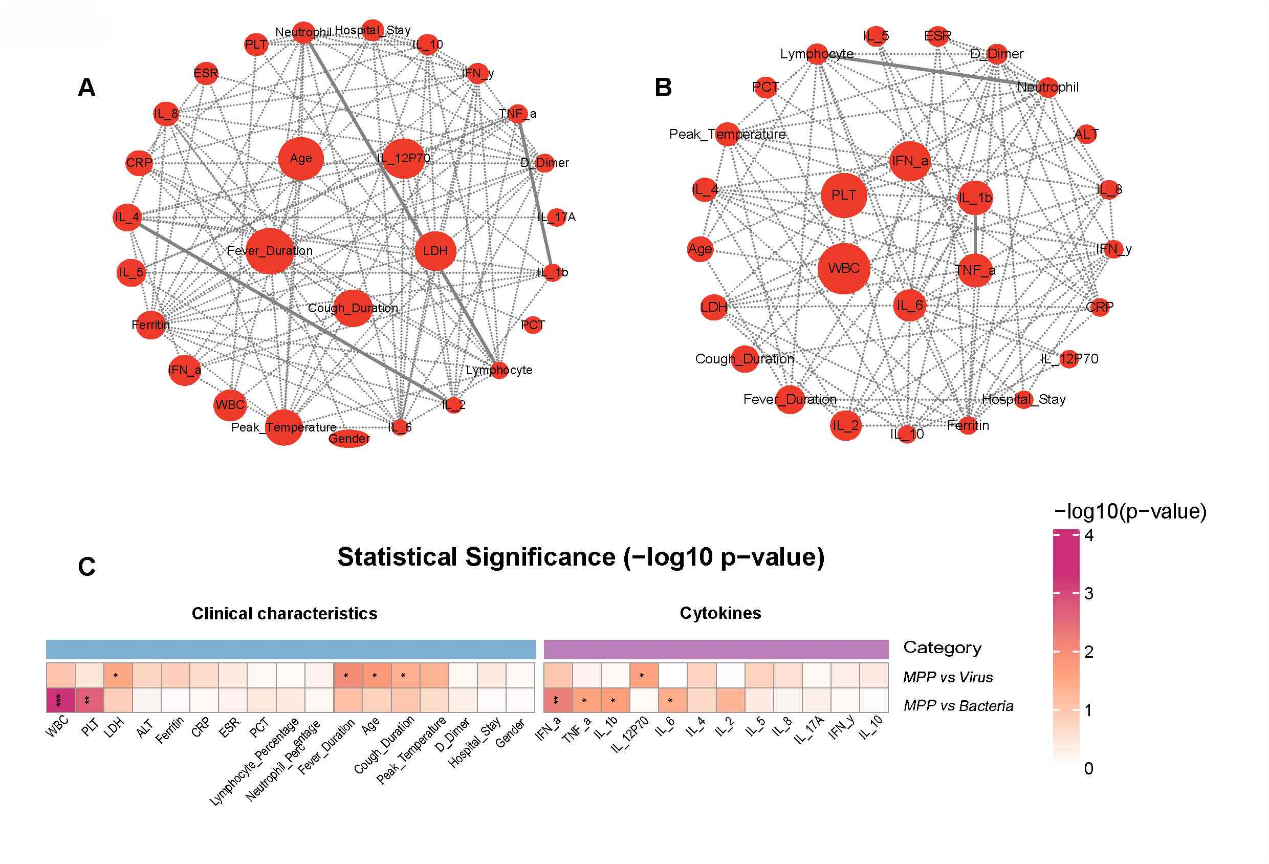 |
| --- |
| **Supplementary Figure S1** Correlation network and statistical analysis of clinical parameters. (a, b) Correlation networks between parameters in different infection groups. (c) Statistical significance heatmap of parameters categorised by clinical characteristics and cytokines (**P* < 0.05, ***P* < 0.01, ****P* < 0.001)  IL, interleukin; IFN, interferon; TNF, tumour necrosis factor; WBC, white blood cell; CRP, C-reactive protein; ESR, erythrocyte sedimentation rate; PLT, platelet; ALT, alanine aminotransferase; LDH, lactate dehydrogenase; PCT, procalcitonin; MPP, *Mycoplasma pneumoniae* pneumonia |

| 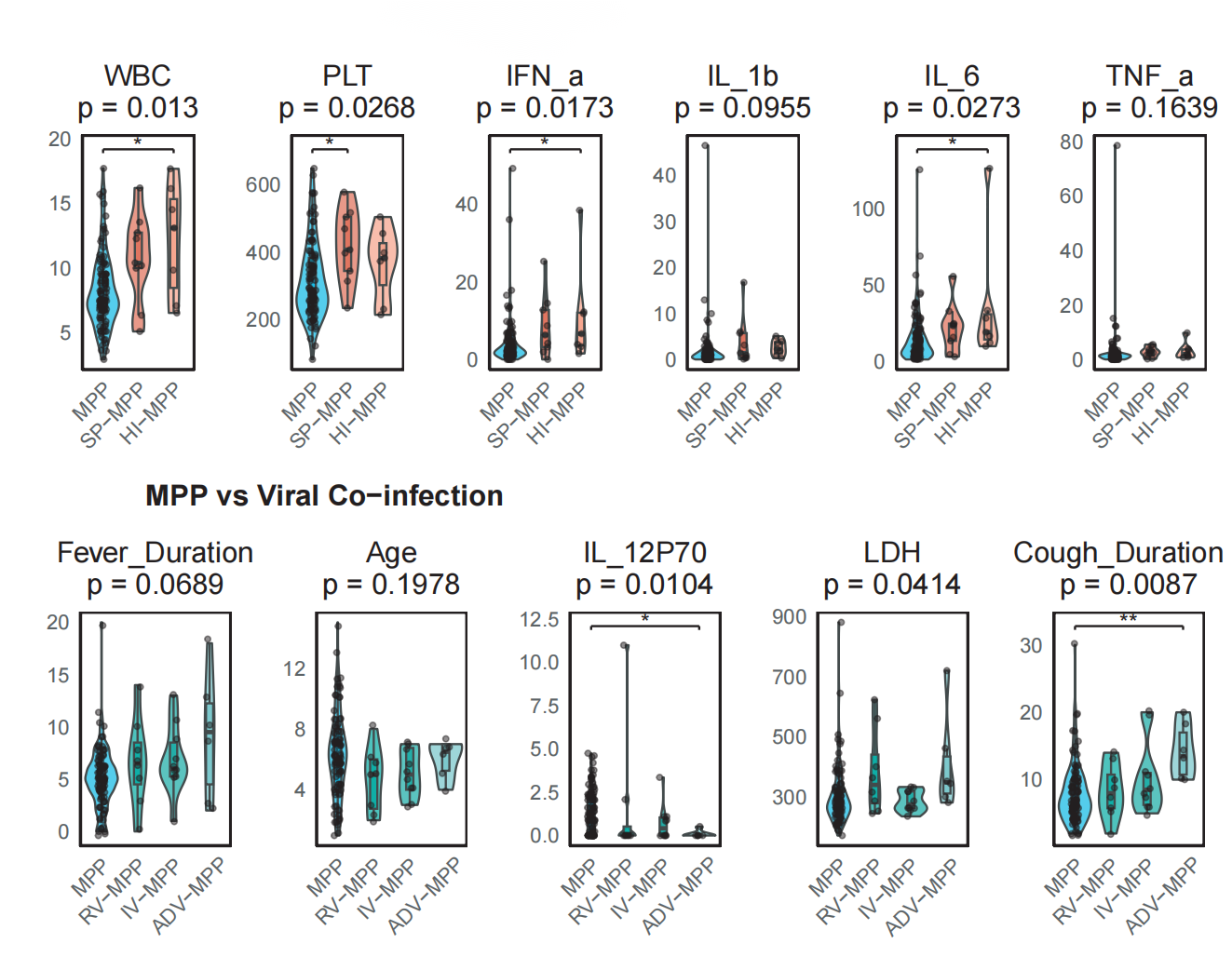 |
| --- |
| **Supplementary Figure S2** Subgroup analysis based on specific pathogens. Violin plots demonstrating differences in key parameters among bacterial subgroups (SP-MPP, HI-MPP) and viral subgroups (RV-MPP, IV-MPP, ADV-MPP) compared to MPP alone (**P* < 0.05, ***P* < 0.01)  WBC, white blood cell; PLT, platelet; IFN, interferon; IL, interleukin; TNF, tumour necrosis factor; MPP, *Mycoplasma pneumoniae* pneumonia; LDH, lactate dehydrogenase; SP, streptococcus pneumoniae; HI, haemophilus influenzae; RV, rhinovirus; IV, influenza virus; ADV, adenovirus |

**Supplementary Table S1** Machine learning results

| Comparison | Model | AUC (95% CI) | Cutoff | Sensitivity | Specificity |
| --- | --- | --- | --- | --- | --- |
| MPP vs. virus | RF | 0.733 (0.605–0.861) | 0.332 | 0.692 | 0.741 |
|  | SVM | 0.727 (0.594–0.861) | 0.438 | 0.5 | 0.948 |
|  | LightGBM | 0.753 (0.636–0.870) | 0.285 | 0.769 | 0.655 |
| MPP vs. bacteria | RF | 0.827 (0.720–0.934) | 0.162 | 0.941 | 0.621 |
|  | SVM | 0.868 (0.772–0.964) | 0.23 | 0.765 | 0.862 |
|  | LightGBM | 0.842 (0.747–0.937) | 0.188 | 0.941 | 0.672 |

MPP, *Mycoplasma pneumoniae* pneumonia; AUC, area under the curve; CI, confidence interval; RF, random forest; SVM, support vector machine; LightGBM, Light Gradient Boosting Machine

**Supplementary Materials**

**Supplementary Figure S1.** Correlation networks and significance heatmap. (a, b) Correlation networks between parameters in different infection groups. (c) Significance heatmap of parameters categorised by clinical characteristics and cytokines (*P* < 0.05, *P* < 0.01, *P* < 0.001).

**Supplementary Figure S2.** Subgroup analysis by pathogens. Violin plots showing differences in key parameters among bacterial subgroups (SP-MPP, HI-MPP) and viral subgroups (RV-MPP, IV-MPP, ADV-MPP) compared with MPP alone.

**Supplementary Table S1.** Detailed machine learning metrics for each model (AUC with 95% CI, sensitivity, specificity, and cutoff values).

**Abbreviations:** MPP, *Mycoplasma pneumoniae* pneumonia; SP, *Streptococcus pneumoniae*; HI, *Haemophilus influenzae*; RV, rhinovirus; IV, influenza virus; ADV, adenovirus; WBC, white blood cell; PLT, platelet; LDH, lactate dehydrogenase; IFN, interferon; IL, interleukin; TNF, tumour necrosis factor; AUC, area under the curve; CI, confidence interval.
